# Changes in Endosymbiont Complexity Drive Host-Level Compensatory Adaptations in Cicadas

**DOI:** 10.1128/mBio.02104-18

**Published:** 2018-11-13

**Authors:** Matthew A. Campbell, Piotr Łukasik, Mariah C. Meyer, Mark Buckner, Chris Simon, Claudio Veloso, Anna Michalik, John P. McCutcheon

**Affiliations:** a Division of Biological Sciences, University of Montana, Missoula, Montana, USA; b Department of Ecology and Evolutionary Biology, University of Connecticut, Storrs, Connecticut, USA; c Department of Ecological Sciences, University of Chile, Santiago, Chile; d Institute of Zoology and Biomedical Research, Jagiellonian University, Krakow, Poland; University of Hawaii at Manoa

**Keywords:** cell biology, endosymbionts, evolution, microscopy

## Abstract

For insects that depend on one or more bacterial endosymbionts for survival, it is critical that these bacteria are faithfully transmitted between insect generations. Cicadas harbor two essential bacterial endosymbionts, “*Candidatus* Sulcia muelleri” and “*Candidatus* Hodgkinia cicadicola.” In some cicada species, *Hodgkinia* has fragmented into multiple distinct but interdependent cellular and genomic lineages that can differ in abundance by more than two orders of magnitude. This complexity presents a potential problem for the host cicada, because low-abundance but essential *Hodgkinia* lineages risk being lost during the symbiont transmission bottleneck from mother to egg. Here we show that all cicada eggs seem to receive the full complement of *Hodgkinia* lineages, and that in cicadas with more complex *Hodgkinia* this outcome is achieved by increasing the number of *Hodgkinia* cells transmitted by up to 6-fold. We further show that cicada species with varying *Hodgkinia* complexity do not visibly alter their transmission mechanism at the resolution of cell biological structures. Together these data suggest that a major cicada adaptation to changes in endosymbiont complexity is an increase in the number of *Hodgkinia* cells transmitted to each egg. We hypothesize that the requirement to increase the symbiont titer is one of the costs associated with *Hodgkinia* fragmentation.

## Abstract (precis)

Sap-feeding insects critically rely on one or more bacteria or fungi to provide essential nutrients that are not available at sufficient levels in their diets. These microbes are passed between insect generations when the mother places a small packet of microbes into each of her eggs before it is laid. We have previously described an unusual lineage fragmentation process in a nutritional endosymbiotic bacterium of cicadas called *Hodgkinia*. In some cicadas, a single *Hodgkinia* lineage has split into numerous related lineages, each performing a subset of original function and therefore each required for normal host function. Here we test how this splitting process affects symbiont transmission to eggs. We find that cicadas dramatically increase the titer of *Hodgkinia* cells passed to each egg in response to lineage fragmentation, and we hypothesize that this increase in bacterial cell count is one of the major costs associated with endosymbiont fragmentation.

## INTRODUCTION

Many organisms associate with microbial symbionts, in interactions that range from transiently pathogenic to stably beneficial from the host perspective. Beneficial symbionts can influence host biology in a variety of ways, but they often confer protection from natural enemies or provide nutrients to their hosts (1–7). Sap-feeding insects harbor obligate endosymbionts that supplement essential nutrients needed for normal host development and reproduction (1, 8–11). For example, cicadas feed exclusively on nutritionally poor plant xylem sap (12, 13), and therefore require supplementation with essential amino acids and vitamins (14). In many of the cicada species characterized to date (but see reference 15), these nutritional services are provided by two transovarially transmitted bacterial endosymbionts, “*Candidatus* Sulcia muelleri” (here referred to as *Sulcia*) and “*Candidatus* Hodgkinia cicadicola” (here *Hodgkinia*) (16–18). We have previously shown that in two cicada genera, *Tettigades* and *Magicicada*, *Hodgkinia* has undergone an unusual form of lineage splitting (19–22). In some of these cicada species, the ancestral single *Hodgkinia* lineage has split into two or more derived lineages, each containing only a subset of the original gene set. These reduced *Hodgkinia* genomes exist in separate cells and are in many cases complementary and partially nonredundant. This complementary gene retention pattern was particularly clear in the genus *Tettigades*, where all characterized genomes contain unique genes from amino acid and vitamin biosynthesis pathways and, thus, all lineages are required to produce the same set of nutrients as the ancestral unsplit genome (19, 22). The number of *Hodgkinia* lineages varies in different cicada species. For example, a species in the cicada genus *Diceroprocta* has one *Hodgkinia* lineage (23), species in the genus *Tettigades* have between one and six *Hodgkinia* lineages (19, 22), and the seven species in the long-lived periodical genus *Magicicada* contain more, possibly dozens of, *Hodgkinia* lineages (20, 21).

A critical aspect of many symbiotic relationships is the transmission of symbionts between host generations. Some organisms acquire symbionts from the environment each generation (24–26), while others have evolved mechanisms to transmit their symbionts directly to their offspring (11, 27–32). We previously speculated that increases in *Hodgkinia* complexity might present intergenerational transmission problems for cicadas (20). As the number of *Hodgkinia* lineages increases, these lineages can start to vary in abundance by more than 100-fold in a single cicada (22). There is therefore a risk to the host of losing the least abundant *Hodgkinia* lineages—which in some cases contain genes essential to *Hodgkinia*’s nutritional functions—if sufficient numbers of *Hodgkinia* cells are not transmitted to each egg. While cicadas could employ several mechanisms to cope with these changes, we have hypothesized that cicadas with more complex *Hodgkinia* populations might compensate by increasing the overall number of *Hodgkinia* cells transmitted to each egg (20). In contrast, we would not expect to see the same pattern for *Hodgkinia*’s partner symbiont, *Sulcia*, which has not been reported to increase in complexity. Finally, little is known about the mechanism of endosymbiont transfer in cicadas outside work from the early 1900s, and nothing is known about how changes in *Hodgkinia* complexity may affect this process. Here we combine modeling, amplicon sequencing, and microscopy across cicada species and populations to describe how increasing endosymbiont complexity affects symbiont transmission in cicadas.

## RESULTS

### Simulating the change to *Hodgkinia* cell transmission numbers.

**FIG 1:**
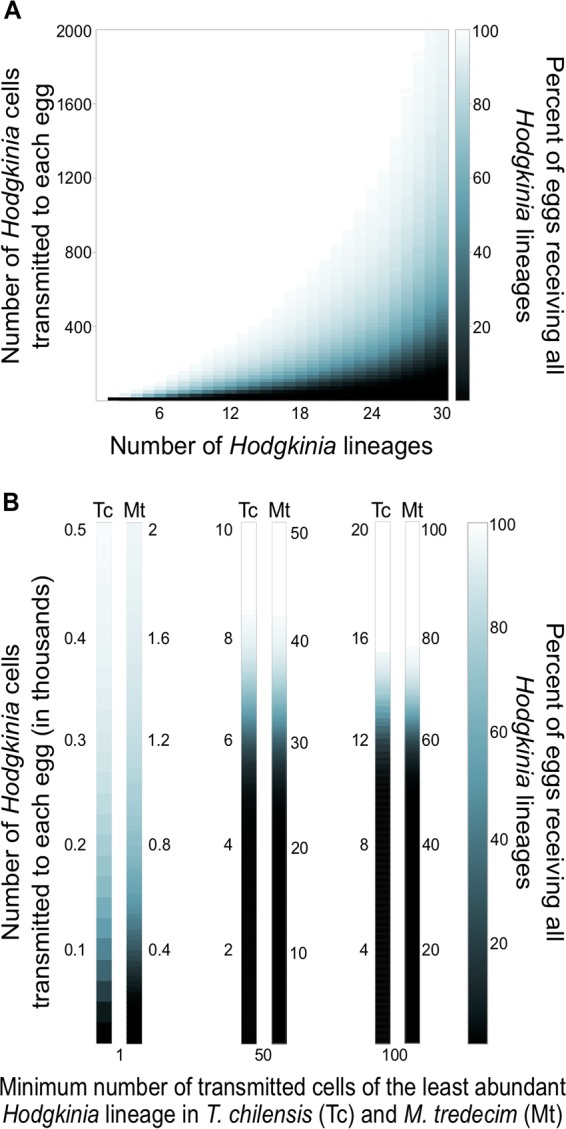
Simulation of the number of *Hodgkinia* cells required to be transmitted with increasing *Hodgkinia* complexity. (A) Proportions of eggs receiving all *Hodgkinia* lineages for a given number of cells transmitted. Values for the abundance of the lineages were taken from sequencing coverages of the finished genomic circles in M. tredecim in reference 21. (B) The same simulation for the six cellular lineages in T. chilensis (Tc, left bar in each pair) and approximately 30 lineages in M. tredecim (Mt, right bar in each pair), requiring one (left), 50 (middle), or 100 (right) cells of the least abundant cellular lineage to be present in all eggs.

We first explored how changes in *Hodgkinia* complexity might affect the number of *Hodgkinia* cells transmitted from mother to egg from a theoretical perspective. Using computer simulations, we modeled transmission by first assuming that *Hodgkinia* lineages are transmitted from mother to egg randomly and that only a single cell of each *Hodgkinia* type is required for egg survival. Figure 1A shows the results for hypothetical cicadas harboring between one and thirty *Hodgkinia* lineages, with relative abundances based on the relative coverage values of completed genomic circles in the M. tredecim assembly (21). We find that as the *Hodgkinia* population becomes more complex, and especially as relative lineage abundances become more uneven, the minimum number of cells required so that all eggs are guaranteed to receive all *Hodgkinia* lineages grows quickly, by more than 2,000-fold. We suspect that a 2,000-fold increase is an upper bound on the changes we might expect to see, since we assume here that cicada eggs are viable if they only transmit one cell of any given lineage to each egg. Nevertheless, these results suggest that we could see up to orders-of-magnitude changes in *Hodgkinia* cell number transmission across a diversity of cicadas hosting *Hodgkinia* communities of various complexities.

We then asked how the total number of *Hodgkinia* cells transmitted to each egg would change if multiple cells of each lineage are needed for its survival. We modeled transmission in cicadas where a minimum of 1 single cell of each lineage was required in all eggs (Fig. 1B, left), 50 cells of each *Hodgkinia* lineage were required (Fig. 1B, middle), and 100 cells of each *Hodgkinia* lineage were required (Fig. 1B, right). These simulations used the *Hodgkinia* complexity of T. chilensis (6 lineages with a 69-fold abundance range) as well as M. tredecim (30 putative lineages with a 74-fold abundance range). For T. chilensis, requiring a single cell of each *Hodgkinia* lineage would necessitate that more than 500 *Hodgkinia* cells were transmitted to each egg. Requiring 50 cells of each *Hodgkinia* lineage would require that more than 8,000 cells are transmitted to each egg, and requiring 100 cells of each lineage would require over 15,000 *Hodgkinia* cells be transmitted to each egg. In each case, for a cicada resembling M. tredecim, the host would need to transmit between 4- and 5-fold more *Hodgkinia* cells than in T. chilensis. These results suggest that we might expect approximately five times more *Hodgkinia* cells transmitted in M. tredecim than T. chilensis.

### Cicadas harboring complex *Hodgkinia* populations transmit more *Hodgkinia* cells to eggs, but not more *Sulcia* cells.

**FIG 2:**
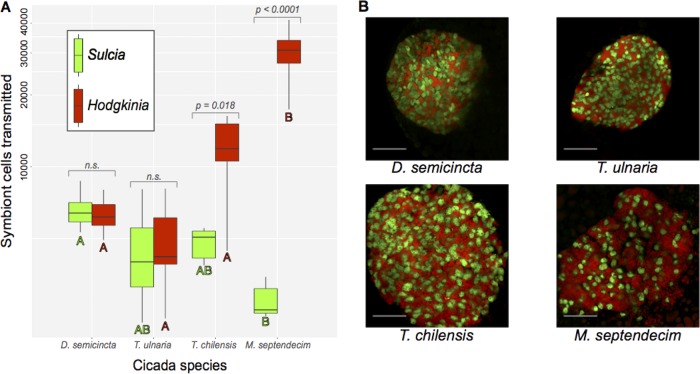
Numbers of symbiont cells transmitted to eggs in different cicadas. (A) Boxplot of the number of *Sulcia* (green) and *Hodgkinia* (red) cells transmitted to eggs in D. semicincta (one lineage, *n* = 5), T. ulnaria (one lineage, *n* = 6), T. chilensis (six lineages, *n* = 6), and M. septendecim (many lineages, *n* = 6). The *y* axis uses a logarithmic scale. Letters above each bar show which values for *Sulcia* (green) and *Hodgkinia* (red) are statistically different from each other based on Tukey’s HSD. Reported *P* values correspond to the test of whether more *Hodgkinia* than *Sulcia* cells are transmitted within a single species. (B) Example images of the symbionts inside the eggs for the same four cicada species. Scale bars represent 50 μm, and the vertical error bars represent the range of calculated cell counts.

Our simulations show that the number of *Hodgkinia* cells transmitted to eggs is likely to increase with increasing *Hodgkinia* complexity. We tested this prediction by estimating the number of *Hodgkinia* cells transmitted to recently laid eggs from various cicada species (Fig. 2). We studied two distantly related cicada species with a single *Hodgkinia* lineage (D. semicincta and T. ulnaria), a species with six *Hodgkinia* lineages (T. chilensis), and a species with perhaps dozens of *Hodgkinia* lineages (M. septendecim). Using fluorescence microscopy, we first counted all of the *Hodgkinia* and *Sulcia* cells from a single confocal image slice. We then counted the number of *Sulcia* cells in a box of known volume and, modeling the symbiont ball as either a perfect sphere or spheroid, estimated the number of *Sulcia* cells in the entire symbiont ball. We then used the counted ratio of *Sulcia* to *Hodgkinia* to estimate the number of *Hodgkinia* cells present in the entire symbiont ball in the egg. We first compared the numbers of *Sulcia* cells transmitted, and found that the average number of *Sulcia* cells transmitted to each egg varies approximately 2-fold across all species, ranging from 2,572 in M. septendecim to 5,643 in D. semicincta. The *Sulcia* cell counts were significantly different (*P* = 0.0005, green labels in Fig. 2A) only between M. septendecim and D. semicincta, but not in other pairwise comparisons. In contrast, the numbers of *Hodgkinia* cells transmitted vary by as much as 6-fold in different cicada species, from 4,889 in T. ulnaria to 30,154 in M. septendecim. The *Hodgkinia* cell count was higher in M. tredecim than in any other species (*P* < 0.001, red labels in Fig. 2A), but the differences in other pairwise comparisons were not significant. Within a cicada, the number of *Hodgkinia* cells differs significantly from *Sulcia* in T. chilensis (Bonferroni-corrected *P* = 0.018) and M. septendecim (*P* < 0.0001), but not in D. semicincta or T. ulnaria. The transmitted *Hodgkinia*/*Sulcia* cell number ratio varies from ∼1:1 in the cicadas with a single *Hodgkinia* lineage, to 2.4:1 in the species with six lineages, to 11.2:1 in the species harboring among the most complex *Hodgkinia* populations known (Fig. 2B).

We estimated the number of transmitted cells of the least abundant *Hodgkinia* lineage by combining these total *Hodgkinia* cell count estimates with our simulation data. Our simulations show that for T. chilensis to transmit 50 cells of the least abundant lineage, it would need to transmit between 8,000 and 9,000 total *Hodgkinia* cells, while for it to transmit 100 cells of the least abundant lineage it would need to transmit close to 16,000 total cells. We find that T. chilensis transmits approximately 12,000 *Hodgkinia* cells on average, and so we would expect it to transmit between 50 and 100 cells of the least abundant lineage. Using the same logic for M. septendecim, which transmits approximately 30,000 total *Hodgkinia* cells (and again assuming all finished circles from reference 21 exist in different cells), we would expect fewer than 50 cells of the least abundant *Hodgkinia* lineage to be present in each M. septendecim egg.

### Cicada eggs seem to receive all *Hodgkinia* lineages, but variation in lineage abundances exists in the cicada population.

Having shown that cicadas can adjust the number of symbiont cells transmitted to their eggs (Fig. 2), we next sought to measure how reliably *Hodgkinia* lineages are transmitted between mother and eggs. We targeted protein-coding genes using amplicon sequencing to measure the differences in cell type abundances in eggs and in the bacteriome tissue of adult cicadas. For two *Tettigades* species, T. chilensis (6 cellular lineages) and T. limbata (5 cellular lineages), the target gene was RNA polymerase subunit B (*rpoB*), which is retained by all cellular lineages in all studied *Tettigades* species (22). Based on metagenomic data for single individuals (in the case of T. chilensis, from a divergent population), *rpoB* variants present in a cicada can vary by as much as 114-fold (22). In *Magicicada* species, gene targets were more difficult to choose because most assembled genomic circles contained few genes and no single gene is universally conserved on each genome (21). We chose to target the electron transfer flavoprotein-ubiquinone oxidoreductase gene (*etfD*), which has two distinguishable gene homologs present at a 6-fold difference in abundance in M. septendecim (21).

We first assessed whether gene abundance estimates generated from amplicon sequencing were consistent between sequencing reactions and with genome abundance estimates we previously generated from metagenomics (21, 22). We compared the abundance estimates for the two methods in three cicada species, and found that, in general, the abundance estimates of genotypes obtained through amplicon sequencing were similar but not exactly the same as those found using metagenomics (see Fig. S1A in the supplemental material). In some cases, abundance estimates were very close (T. chilensis), while in others there was significant deviation in the relative abundance estimates for some lineages (T. auropilosa and T. limbata). Given that our genomic libraries were prepared using PCR-free methods or with <10 PCR cycles, and that our amplicon approach always required multiple (>25 in total) rounds of PCR with primers that might cause bias against some template variants, we assume that the proportions found using metagenomics are more accurate. Nevertheless, the abundance estimates found using amplicon data were consistent among technical replicates of the same sample (Fig. S1A) as well as between different parts of the bacteriome tissue from the same individual cicada (biological replicates, Fig. S1B), giving us confidence that the abundance differences we find between individuals result from genuine biological variation rather than methodological artifacts.

10.1128/mBio.02104-18.1 FIG S1 Testing the bias in amplicon sequencing experiments. (A) Technical replicates: the relative abundance of *rpoB* genotypes in replicate amplicon libraries prepared from DNA samples that had previously been used for metagenomic sequencing, compared to metagenomics-based estimates. In the case of *T. limbata*, the metagenomic library was prepared using a PCR-free protocol. (B) Biological replicates: the relative abundance of *Hodgkinia rpoB* genotypes in individual bacteriome lobes, versus all remaining lobes pooled, in three *Tettigades chilensis* specimens from a single population. Download FIG S1, TIF file, 3.2 MB. Copyright © 2018 Campbell et al. 2018 Campbell et al. This content is distributed under the terms of the Creative Commons Attribution 4.0 International license.

**FIG 3:**
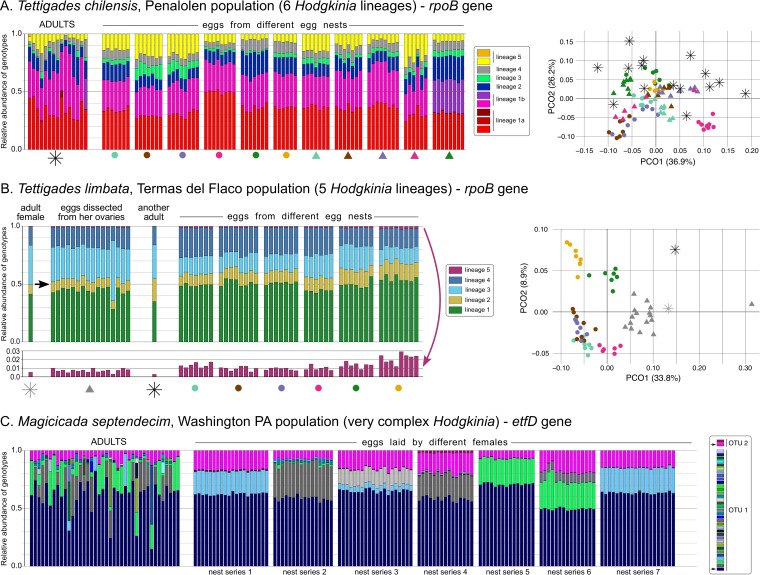
The relative abundances of *Hodgkinia* variants within populations of three cicada species, based on amplicon sequencing of symbiont-carried protein-coding genes. For replicate adults and batches of eggs laid by individual females (egg nests), we plotted the relative abundance of *Hodgkinia rpoB* genotypes that correspond to six or five recognized lineages (*Tettigades* spp. [A and B]) or of *Hodgkinia etfD* genotypes whose nature is less clear (M. septendecim [C]). The relationships among samples of the two *Tettigades* species, based on the relative abundance of lineages rather than genotypes, is presented on principal component analysis plots; shapes correspond to those shown below groups of bar plots. In panel B, in a plot where scale is 10× magnified, we additionally show how the relative abundance of the rare lineage 5 varies among samples. In panel C, unique genotypes within the two observed OTUs are shown in shades of blue/green/gray (OTU1) or pink (OTU2), and those genotypes that are found in all samples are indicated with arrows on the legend.

Our amplicon data revealed sequence complexity that was not detected in our previous metagenomic results (21, 22). In Tettigades limbata, all specimens host the same *rpoB* genotypes that exactly correspond to sequences from our previous metagenomics work (22). The same is true in T. chilensis, except that in some cases one genotype has been replaced or complemented by another that differs by one nucleotide (Fig. 3A). In the case of M. septendecim, all sampled adults and eggs hosted two *Hodgkinia etfD* genotypes that were 6.7% divergent from each other at the nucleotide level (Fig. 3C). However, both amplicon sequences differed by one nucleotide substitution from the previously annotated *etfD* homologs in a metagenomic assembly of M. septendecim from a different brood (21). We suspect that these differences likely correspond to different alleles of the same *etfD* homologs. Additionally, all M. septendecim specimens hosted several genotypes that were less than 1% divergent from one of the two universally prevalent homologs (OTUs 1 and 2 in Fig. 3C). However, none of these derived genotypes are present in all samples, and all adults and egg nests harbor different combinations of these derived genotypes.

We next tested whether cicadas reliably transmit all *Hodgkinia* lineages to each egg, and measured how the proportion of endosymbiont lineages varies among eggs laid by a single female and within populations of single cicada species. Based on our simulation (Fig. 1) and cell count data (Fig. 2), we suspected that some cicada eggs might not receive all *Hodgkinia* lineages. Our amplicon data did not support this suspicion: we find that all *Tettigades* eggs contain all *rpoB* genotypes (Fig. 3A and B), and in *Magicicada*, all eggs contain both universally prevalent *etfD* genotypes (Fig. 3C). We then compared the variation in lineage proportions among adult cicadas, and among batches of eggs laid by the females in the same populations. In principal component analysis, T. chilensis eggs from the same nest tended to cluster together, separately from eggs from other nests, and the ADONIS test revealed significant differences in proportions of *Hodgkinia* lineages among eggs from the eleven characterized nests (F_10,68_ = 33.88, *P* < 0.001) (Fig. 3A). In T. limbata, the differences in the proportions of lineages were less striking, but also significant among the six sampled egg nests (F_5,37_ = 30.16, *P* < 0.001) (Fig. 3B). These differences were partly driven by the variable relative abundance of the least common lineage 5, which ranged among the studied samples over 10-fold (between 0.25% and 2.72%) (Fig. 3B).

We note that in M. septendecim, a large number of unique genotypes complicates lineage abundance comparisons among samples. However, the comparisons of the relative abundance of the two universally prevalent *etfD* homologs revealed highly significant differences between egg batches from different females (GLM; genotype from OTU 1: F_6,119_ = 274.1, *P* < 0.001; genotype from OTU 2: F_6,119_ = 140.0, *P* < 0.001). We suspect that this sequence variation is the result of cicada population subdivision as well as some ancestral polymorphism in the cicada populations. There is some support for ancestral polymorphism in *Magicicada*: comparing the *etfD* genotype composition in individuals from different broods indicates that some of the variation is ancient and was present in the common ancestors of different broods (Fig. S2). Overall, the variation in lineage abundances that exists within cicada populations suggests that these insects can tolerate a relatively wide range of *Hodgkinia* lineage abundances. Individual mothers, however, seem to avoid substantial genotype abundance shifts between generations when transmitting symbionts to their offspring, at least in the single generation we measured here.

10.1128/mBio.02104-18.2 FIG S2 The relative abundances of *Hodgkinia* genotypes among adult *Magicicada septendecim* specimens from different broods, based on *etfD* amplicon data. Only the genotypes represented by at least 1% of reads in at least one library were used for the relative abundance calculations. Bars indicated as “low abund.” represent the cumulative abundance of genotypes in each of the two OTUs that were represented by more than 1% of reads in at least one library but never by more than 10% of reads. Download FIG S2, TIF file, 1.0 MB. Copyright © 2018 Campbell et al. 2018 Campbell et al. This content is distributed under the terms of the Creative Commons Attribution 4.0 International license.

### The cell biological mechanism of symbiont transmission in cicadas is (mostly) conserved.

**FIG 4:**
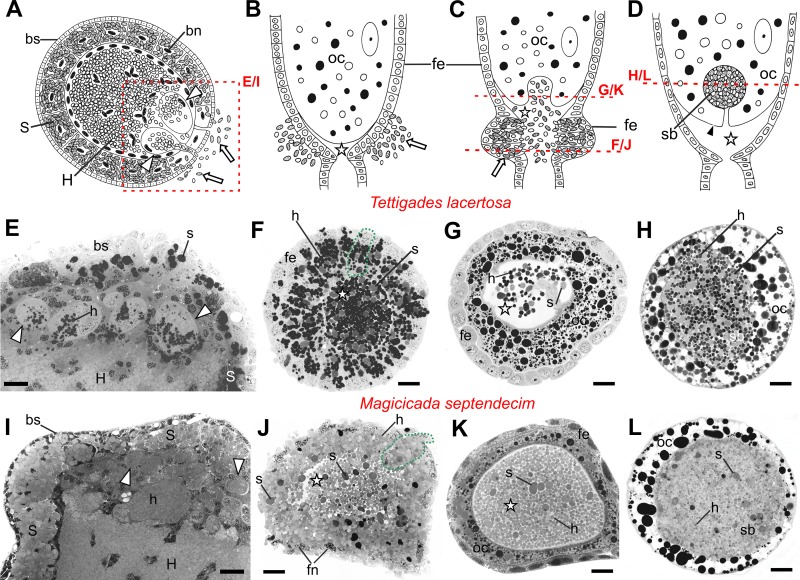
Transovarial transmission of endosymbiotic bacteria between cicada generations. (A to D) Schematic representation of the successive stages of transmission, including the emigration of symbiont cells from the bacteriome (A), their migration through follicular epithelium into the perivitelline space of an ovariole (B and C), and then into an invagination within the basal part of the terminal oocyte (C) where they form a “symbiont ball” (D). The microphotographs of methylene blue-stained sections indicated with a red box or red line on the schematics are shown for two cicada species: Tettigades lacertosa, which hosts three *Hodgkinia* lineages (E to H), and Magicicada septendecim, which hosts very complex *Hodgkinia* (I to L). The overall transmission process appears the same in both species, but the numbers of migrating bacterial cells appear much greater in *Magicicada*. We note that the relative intensity of the symbiont cell staining varies depending on species and their physiological state, and that the staining is consistently higher in cells undergoing migration. This has been observed in other hemipteran symbioses (39, 40), and may be due to changes in methylene blue-attracting protein concentrations or distributions during this phase. S, bacteriocyte with *Sulcia*; bn, bacteriocyte nucleus; bs, bacteriome sheath; H, syncytium with *Hodgkinia* cells; fe, follicular epithelium; fn, follicular cell nucleus; oc, oocyte; sb, symbiont ball; s, *Sulcia* cell; h, *Hodgkinia* cell; white arrow, symbiotic bacterium; white arrowheads, *Hodgkinia*-carrying vesicles within syncytium; encircled with green, dotted line, follicular cell filled with symbiotic bacteria; white star, perivitelline space; black arrowhead, oocyte membrane. Scale bar, 50 μm.

Because we found a clear adaptation by hosts in terms of changing the number of symbionts transferred in cicadas with various levels of *Hodgkinia* complexity (Fig. 2), we wondered whether we could also observe changes to the mechanism of symbiont transfer. At the resolution of light microscopy, we find that the mechanism of endosymbiont transfer does not differ between T. lacertosa and M. septendecim, nor does it differ significantly from what Paul Buchner described in an unidentified African cicada species which appeared to harbor *Sulcia* and *Hodgkinia* (33) (Fig. 4). More generally, at this resolution, the mode of symbiont transmission appears well conserved throughout auchenorrhynchan insects (18, 34). In mature cicada females, *Hodgkinia* and *Sulcia* cells are released from separate regions of the bacteriome into the hemolymph (Fig. 4A). Notably, *Hodgkinia* emigrates through large, nucleated subcellular compartments that form within the syncytium where it normally resides, while *Sulcia* is released directly from peripheral bacteriocytes. Subsequently, both bacterial symbionts are transported toward the ovarioles and through follicular cells into the perivitelline space (Fig. 4B and C). As the number of symbionts in that space increases, the oocyte membrane creates a deep invagination where the symbionts gather. Later, as the opening closes, the intermixed *Sulcia* and *Hodgkinia* cells form a characteristic “symbiont ball” in each egg (Fig. 4D).

The transmission process does not appear to be qualitatively different between *Tettigades* (Fig. 4E to H) and *Magicicada* (Fig. 4I to L). However, consistent with our fluorescence microscopy observations (Fig. 2A), in *Magicicada* the overall number of bacterial cells transmitted to the oocyte is visibly higher than in *Tettigades*, and the ratio of *Hodgkinia* cells to *Sulcia* cells is higher than in *Tettigades* (Fig. 2B). Together, these data indicate that in response to *Hodgkinia* splitting, cicadas have adjusted their ancient transmission pathway to increase the numbers of transmitted *Hodgkinia* cells, but not *Sulcia* cells.

## DISCUSSION

### Cicadas adapt to increases in *Hodgkinia* complexity.

The strong selective pressure to reliably transmit nutritional symbionts to offspring is reflected in a conserved mechanism for transmission in cicadas. In D. semicincta and T. ulnaria, cicada species diverged by tens of millions of years (35–38), both *Sulcia* and *Hodgkinia* have stable, conserved genomes (19, 23), and we have shown here that these two cicadas also transmit similar numbers of *Hodgkinia* and *Sulcia* cells to each egg (Fig. 2A). Within the last ∼4 million years, *Hodgkinia* in some *Tettigades* species has become more complex due to lineage splitting and genome reduction (19, 22). This same process had led to the incredibly complex situation seen in all *Magicicada* species, which we estimate has been ongoing over the last 5 to 20 million years (21).

This increase in symbiont complexity could pose a problem for the cicada. Rather than a single lineage each of *Sulcia* and *Hodgkinia*, cicadas with more complex *Hodgkinia* have *Sulcia* plus many distinct—but still essential—*Hodgkinia* lineages that must be transmitted together for the cicada’s offspring to survive. This problem has three potential and not mutually exclusive solutions. Solution 1: the host evolves a mechanism to distinguish between *Hodgkinia* lineages and actively places all lineages into each egg. Because even the largest *Hodgkinia* genome no longer encodes the machinery to make its own membranes, the host must define *Hodgkinia*’s envelope, so this solution is formally possible. Solution 2: the host could increase the number of *Hodgkinia* cells transmitted to each egg, thereby increasing the odds that lower-abundance lineages make it to each egg. Solution 3: the host mother could produce some proportion of (presumably inviable) eggs that do not receive all *Hodgkinia* lineages. This last option is likely to come with a negative fitness cost for the host.

We currently do not have the ability to measure whether hosts actively select certain *Hodgkinia* lineages (solution 1). We do find that cicadas seem to be able to tolerate substantial variation in *Hodgkinia* lineage abundances (Fig. 3), suggesting that if a host selection process does happen then it is not highly accurate over cicada generations. We find clear evidence that hosts increase the number of *Hodgkinia* cells transmitted to eggs (solution 2, Fig. 2), but no evidence that any egg is missing any *Hodgkinia* lineages (solution 3, Fig. 3). From these data, we conclude that increasing the symbiont transmission number is likely the key adaptation by the cicada to compensate for *Hodgkinia*’s increasing complexity. The increase in *Hodgkinia* transmission numbers appears to solve this aspect of the symbiont complexity problem, since all cellular lineages seem to be reliably transmitted to all offspring (Fig. 3) We note, however, that it is possible that some low-abundance lineages are occasionally lost in certain eggs and that we lack the sensitivity to detect it.

Individual *Hodgkinia* lineages can differ in abundance more than 100-fold in adult cicadas (22). Since eggs receive similar proportions of the lineages that were present in their mother (Fig. 3), the least abundant lineages will be the primary drivers of the required increase in the number of transmitted *Hodgkinia* cells. Because it seems unlikely that cicadas can indefinitely increase the number of *Hodgkinia* cells transmitted to each egg, cicadas must also decrease the number of cells transmitted of the least abundant *Hodgkinia* lineage. According to our simulations, T. chilensis and M. septendecim might receive fewer than 100 cells of the least abundant *Hodgkinia* lineage (Fig. 1). These estimates are consistent with our expectation based on relative sequencing coverage: we estimate that T. chilensis eggs receive only ∼80 cells of the least abundant lineage (based on sequencing coverage for T. chilensis of a different population, where its equivalent comprises 0.8% of the total *Hodgkinia* population [22]), and M. septendecim eggs likely receive fewer than 50 cells of the least abundant lineage.

We find that cicadas with single *Hodgkinia* lineages transmit substantially more *Hodgkinia* cells than strictly necessary (Fig. 2). This “surplus” of transmitted cells might prevent an immediate fitness cost to the host as a result of *Hodgkinia* lineage splitting, and is likely the reason we see only an ∼6-fold increase in *Hodgkinia* cells transmitted as *Hodgkinia* complexity increases, rather than the ∼2,000-fold increase seen in our simulations (Fig. 1A). The relatively smaller increase that we measure empirically (Fig. 2) versus that which we predict computationally (Fig. 1) might also be due to more than one *Hodgkinia* genomic circle sharing cellular lineages (22). Our genomic data strongly suggest that at least in the genus *Tettigades*, some *Hodgkinia* genomic circles are present in the same *Hodgkinia* cell, but we have not yet verified this result using other methods (22). While the reduction of the minimum number of required cells is one method to prevent the required transmission size from spiraling out of control, we also know that lineage splitting in at least some cicadas is ongoing (21). Therefore, the lower cell number distribution limit is not something that can be reduced indefinitely. For example, the cobalamin biosynthesis gene *cobQ* is carried by only 0.8% of all *Hodgkinia* cells in T. chilensis (22), so further decrease in the abundance of the *cobQ*-bearing lineage may negatively affect the supply of this vitamin.

### *Hodgkinia* is driving the adaptation in its host.

Importantly, we have shown that the number of *Sulcia* cells transmitted remains relatively stable in all of the studied cicadas (and may be actually decreasing in *Magicicada* [Fig. 2A]). We thus infer that the principal driver of the transmission changes we show here is specific to *Hodgkinia*-related processes rather than a general change in host transmission strategy. It is also formally possible that *Hodgkinia*’s transmission numbers could have changed before *Hodgkinia* started splitting, and thus be enabling the fragmentation we see in some cicadas. The transmission numbers for *Sulcia* and *Hodgkinia* in cicadas with unsplit *Hodgkinia* lineages are on the high end for transovarially transmitted symbionts estimated for a wide range of other hemipteran insects (Table 1), but this alone seems unlikely to be the main driver of lineage splitting in *Hodgkinia* because some cicadas continue to retain *Hodgkinia* with a single genome structure.

**TABLE 1:** Estimated numbers of endosymbiont cells within symbiont balls in eggs of different emipteran species^*a*^

Host species	Taxonomic position	Symbiont species	No. of cells on symbiont ball cross section	Estimate of symbiont cell no. in the ball	Reference
*Nasonovia* sp.	Sternorrhyncha: Aphidoidea: Aphididae	*Buchnera*	Multiple sections	886 ± 60	30
*Acyrthosiphon pisum*	Sternorrhyncha: Aphidoidea: Aphididae	*Buchnera*	Multiple sections	1,872 ± 524	30
*Uroleucon ambrosiae*	Sternorrhyncha: Aphidoidea: Aphididae	*Buchnera*	Multiple sections	8,223 ± 428	30
*Ceroputo pilosellae*	Sternorrhyncha: Coccomorpha: Pseudococcidae	*Tremblaya phenacola*	20	67–104	A. Michalik, personal communication
*Phenacoccus aceris*	Sternorrhyncha: Coccomorpha: Pseudococcidae	*Tremblaya phenacola*	21	72–111	A. Michalik, personal communication
*Trionymus thulensis*	Sternorrhyncha: Coccomorpha: Pseudococcidae	*Tremblaya princeps*^*b*^	21	72–111	A. Michalik, personal communication
*Greenisca brachypodii*	Sternorrhyncha: Coccomorpha: Eriococcidae	*Kotejella + Arsenophonus*	∼100	750–1,158	41
*Psylla alni*	Sternorrhyncha: Psyllomorpha: Psyllidae	Unknown, two species	64	384–593	40
*Cacopsylla melanoneura*	Sternorrhyncha: Psyllomorpha: Psyllidae	Unknown, two species	46	234–361	40
*Ommatidiotus dissimilis*	Auchenorrhyncha: Fulgoromorpha: Caliscelidae	*Sulcia + Vidania + Sodalis*	81	547–844	39
*Dictyophara europaea*	Auchenorrhyncha: Fulgoromorpha: Dictyopharidae	*Sulcia + Vidania + Sodalis*	∼218	2,416–3,728	A. Michalik, personal communication
*Macrosteles laevis*	Auchenorrhyncha: Cicadomorpha: Cicadellidae	*Sulcia*^*b*^ + *Nasuia*	118	962–1,485	42
*Graphocraerus ventralis*	Auchenorrhyncha: Cicadomorpha: Cicadellidae	*Sulcia* + yeast	135	1,177–1,817	43
*Cicadula quadrinotata*	Auchenorrhyncha: Cicadomorpha: Cicadellidae	*Sulcia* only	56	315–485	43
*Deltocephalus pulicaris*	Auchenorrhyncha: Cicadomorpha: Cicadellidae	*Sulcia + Nasuia*	∼162	1,548–2,388	44
*Jassargus pseudocellaris*	Auchenorrhyncha: Cicadomorpha: Cicadellidae	*Sulcia + Nasuia*	96	706–1,089	67
*Arthaldeus pascuellus*	Auchenorrhyncha: Cicadomorpha: Cicadellidae	*Sulcia + Nasuia*	81	547–844	67
*Centrotus cornutus*	Auchenorrhyncha: Cicadomorpha: Membracidae	Unknown, four species	∼210	2,284–3,525	A. Michalik, personal communication
*Tettigades lacertosa*	Auchenorrhyncha: Cicadomorpha: Cicadidae	*Sulcia + Hodgkinia* (three)	630	11,895–18,314	This study (Fig. 4H)
*Magicicada septendecim*	Auchenorrhyncha: Cicadomorpha: Cicadidae	*Sulcia + Hodgkinia* (complex)	∼1,750	55,071–84,787	This study (Fig. 4L)

a Because bacterial species are sometimes hard to distinguish, cells of different species were counted together. For species other than aphids, the number is based on cell count on a single cross section, with the assumption that the ball was spherical and symbionts evenly distributed within the ball. The lower estimate is based on the assumption that the section was made through the center of a spherical symbiont ball; the higher estimate assumes that the section was made at 25% of the ball length. Note that these estimates may be inaccurate if the section was made even closer to the ball edge, or if the shape of the ball departed significantly from spherical.

b Cells of endosymbionts of two species contain endobacterial symbionts, which were not included in the counts.

Though the increase in *Hodgkinia* transmission number is a solution for the cicadas’ immediate problem, it raises other potential complications. Cicadas, including *Magicicada*, typically lay between 400 and 600 eggs (45–47), but M. septendecim individuals transmit ∼6-fold more *Hodgkinia* cells to each egg than D. semicincta or T. ulnaria individuals. If a cicada is to continue transmitting larger numbers of *Hodgkinia* cells to all eggs, fewer eggs must be laid, its *Hodgkinia* population must be replenished as it lays eggs, or a larger *Hodgkinia* population must be maintained in the adult cicada stage. Laying fewer eggs is likely to lead to fewer offspring and so is unlikely to be favored. It may be possible for cicada mothers to replenish the *Hodgkinia* population as they lay eggs, because Buchner has suggested that *Hodgkinia* may be dividing prior to transmission into eggs (33). However, our microscopy shows no clear evidence of this (Fig. 4), so it is unclear if this is an important mechanism for increasing *Hodgkinia* numbers. This mechanism would also require relatively rapid *Hodgkinia* reproduction since cicadas lay their eggs within a short time span (47). While not definitive, we have also gathered anecdotal evidence that cicadas with more complex *Hodgkinia* populations harbor larger *Hodgkinia* populations as adults (20), but we currently have no solid data on the total number of symbiont cells in adult cicadas. But maintaining a larger *Hodgkinia* population would bring its own complications, as the cicada has to provide more tissue space and nutrients for a larger *Hodgkinia* population, and runs the risk of crowding out its partner symbiont *Sulcia* (Fig. 2) (20).

### Symbiont population sizes could affect host and symbiont levels of selection.

An increase in *Hodgkinia*’s intracicada population size may have implications for the long-term evolution of the symbiosis. As in any endosymbiosis, the evolutionary trajectories of host and symbiont are not inevitably and permanently aligned. For the host, it is important that symbionts are maintained at small effective population sizes, which is often achieved by subjecting symbionts to strong population bottlenecks at transmission (48–51). There are three evolutionary consequences to maintaining small intrahost symbiont effective population sizes. First, it reduces the efficacy of symbiont-level selection for selfish traits, since selection is less efficacious in small populations. Second, small symbiont populations will harbor less diversity, further decreasing the efficacy of symbiont-level selection. Finally, with relatively few symbionts within a cicada, there are fewer mutational targets to acquire the complementary gene loss required for *Hodgkinia* splitting to happen. While speculative, it seems possible that increasing the number of *Hodgkinia* cells transmitted might itself make the splitting process more likely to happen, because it would decrease the level of control that the host can exert on its symbionts. Larger symbiont populations would lead to more intrahost variation, and thus, more chances for lineage splitting by mutation and drift or by symbiont-level cheating as previously hypothesized (19–21). In this scenario, the increasing numbers of *Hodgkinia* cells might lead to a positive-feedback loop, where the compensatory changes cicadas have evolved in response to increasing *Hodgkinia* complexity might themselves make the problem of splitting worse.

It is perhaps unsurprising that symbiont evolution is driving compensatory adaptations in cicadas. There are a number of other examples of what appears to be host compensatory evolution to symbiont change, such as nuclear genes responding to high mitochondrial substitution rates in plants (52, 53) and primates (54), horizontal transfer of bacterial genes to the nucleus to maintain symbiont function in several eukaryotic groups (reviewed in reference 55), and the evolution of trafficking systems to move gene products between host and symbiont (61–63). These examples highlight the pervasiveness of host compensation to the evolution of symbiont traits, and might reflect the peril of critical reliance of hosts on vertically transmitted endosymbionts (64–66). If endosymbionts erode in functionality due to host restriction and genetic drift, the host must compensate somehow—potentially through a shift in host ecology or the replacement of its degrading symbiont (64)—or suffer the consequences of reduced fitness or, in extreme cases, extinction of the entire symbiosis.

## MATERIALS AND METHODS

### Egg simulation protocol.

For each of 1 to 30 hypothetical *Hodgkinia* cell lineages, between 1 and 2,000 *Hodgkinia* cells (in increments of 20) were sampled with replacement and placed in hypothetical eggs that initially had no symbionts present. After all symbionts were placed in eggs, each egg was checked for the presence of each *Hodgkinia* lineage. If at least one cell of every lineage was present in the egg, that egg was determined to be viable. The total proportion of viable eggs was then calculated after 10,000 iterations. This same procedure was repeated for all combinations of lineages and cell numbers. For the T. chilensis and M. tredecim experiments shown in Fig. 1B, the same simulation was performed for 6 and 30 lineages, respectively, but with the requirement that a minimum number of cells (1, 50, or 100) of each lineage be present in a given egg for it to be deemed viable, as described in Results. Python code used for the simulation is available at https://github.com/mattsoup/egg_simulation.

### Sample collection.

Details of samples used for the study are shown in Table S1 in the supplemental material. For both *Tettigades* and *Magicicada* samples, all eggs in an “egg nest” were assumed to be laid by the same female. For *Tettigades* samples, we assumed that different nests were laid by different females because we collected different egg nests on different branches in places where the cicada population density was high. In the case of *Magicicada*, we assumed that a series of adjacent egg nests on a single branch were produced by the same female. We attempted to verify this during data analysis, and as a precaution have removed any nests where eggs contained a different set of *Hodgkinia* genotypes than eggs in other nests in a series under the assumption that these may have been laid by a different female.

10.1128/mBio.02104-18.3 TABLE S1 Sample collection information. Download Table S1, XLSX file, 0.05 MB. Copyright © 2018 Campbell et al. 2018 Campbell et al. This content is distributed under the terms of the Creative Commons Attribution 4.0 International license.

### DNA extraction.

DNA from M. septendecim eggs and adult tissue, as well as *Tettigades* adult tissue, was extracted using a DNeasy Blood and Tissue kit (Qiagen, catalog number 69506). The process of DNA extraction from *Tettigades* eggs was done by lysing the eggs in DNeasy lysis buffer followed by purification using Sera-Mag SpeedBeads (carboxylate-modified particles, Thermo Scientific catalog number 09-981-123).

### Amplicon library preparation.

Amplicon sequencing libraries were prepared following a two-step PCR protocol described in detail previously (22). For the first PCR step, we used primers targeting a gene retained on all (*Tettigades* spp., *rpoB* with primers TCGCTRAGYTTAAYAAACGGATG and ATCGDTATTGCGMRGAGCTT) or some (*Magicicada*, *etfD* with primers ACGTTATTGTGGCYGAAGGTGC and ACGTTATTGTGGCYGAAGGTGC) *Hodgkinia* genomic circles present in a cicada, complete with Illumina adapters. During the second, indexing PCR step, additional adapters and sample-specific barcodes were added. The libraries were roughly quantified by comparison of band brightness following gel electrophoresis, pooled, and sequenced across three Illumina MiSeq lanes, alongside other libraries not included here. Sequencing for *Tettigades* was done across several MiSeq runs at the University of Montana Genomics Core, Missoula, MT. Sequencing for *Magicicada* was done on a MiSeq at the Genetic Resources Core Facility, Johns Hopkins Institute of Genetic Medicine, Baltimore, MD.

### Amplicon data analysis.

The amplicon data were processed using mothur v. 1.39.5 (56). All reads were assembled into contigs, primer sequences were trimmed, and those reads with primer mismatches, ambiguous bases, homopolymer stretches >10 bp, or departing from the expected contig length by more than 10 bases were discarded. We then identified unique genotypes in the resulting filtered data set, producing a table with information on the number of reads representing each genotype in each library. For the two *Tettigades* species, the exact sequences of *Hodgkinia* variants, alongside information on the relationship among and sequence diversity within cellular lineages, were available from our prior work (22). After verifying that no other abundant nonchimeric sequences were present within the table, we used only the counts of these exact genotypes for statistical comparisons. In the case of M. septendecim, we identified all genotypes that made up at least 1% of at least one library. The manual alignment and inspection of the sequences revealed that they represented two 99% OTUs that were about 7% divergent from each other. After manually identifying and discarding chimeras between these two OTUs, we used the count data for the remaining 37 genotypes, which together made up 83.0% of reads in a library on average (range 71.8% to 86.0%), for visualization and analyses.

Statistical comparisons of the lineage abundance among samples were conducted using R version 3.1.3 (57). Principal component analysis was conducted based on Bray-Curtiss dissimilarity matrices (functions vegdist and pco from packages vegan and labdsv, respectively) (58, 59), and the results visualized using ggplot2 function (60). The multivariate analysis of variance among egg nests was conducted using the function adonis (package vegan [58]). The relative abundances of the two universally prevalent *Hodgkinia* genotypes among *Magicicada* egg nests were determined using Generalized Linear Modeling, assuming quasibinomial error structure to account for overdispersion in the data.

### Microscopy.

Fluorescent *in situ* hybridization microscopy using small-subunit rRNA probes was conducted on eggs as described previously for other cicada tissues (19). Briefly, eggs were broken manually, fixed for one hour in Carnoy’s solution, and then incubated in prehybridization solution (12.5% dextran sulfate, 2.5× SCC, 0.25% BSA) at 37°C for 1 h. Eggs were then briefly washed with warm 2× SCC and incubated overnight at 37°C with hybridization solution (prehybridization solution, 10 ng/μl probe, 1.5 μg/μl Hoechst 33258) in a humidity chamber. Eggs were then incubated in 2× SCC at 37°C for 1 h, briefly rinsed with deionized H_2_O, placed on a glass slide, and covered with a cover slip. Probes used were Cy3-CCAATGTGGGGGWACGC for *Sulcia*, Cy5-CCAATGTGGCTGACCGT for *Hodgkinia* in D. semicincta, Cy5-CCAATGTGGCTGRCCGT for *Hodgkinia* in *Tettigades*, and Cy5-CCAATGTGGCTGTYCRT for *Hodgkinia* in M. septendecim. Symbiont balls in eggs were imaged on a Zeiss 880 confocal microscope. The total volume of the ball was estimated as either a sphere or spheroid. The number of *Sulcia* cells was counted within a box of approximately 50 × 50 × 10 μm^3^ within the tissue, and this number was used to estimate the total number of *Sulcia* cells present in the egg. The ratio of *Hodgkinia* to *Sulcia* cells present was then calculated on a single slice, and this value was used to estimate the number of *Hodgkinia* cells present. This process was repeated three times for each sample, and then averaged between samples. Separate ANOVA tests were run (and corrected for multiple comparisons by Bonferroni correction) (i) using *Sulcia* cell number estimates for all species; (ii) using *Hodgkinia* cell number estimates for all species; and (iii) using cell number estimates for both symbionts, separately for each host species. In the first two comparisons, a *post hoc* Tukey HSD test was used to identify species pairs with significantly different symbiont counts.

For light microscopy, partially dissected cicada tissues were fixed in the field and stored in 0.05 M phosphate-buffered solution with 2.5% glutaraldehyde, then fully dissected and postfixed using 1% osmium tetroxide, and embedded in Epon 812 (Serva, Germany) epoxy resin. Semithin sections (1 µm thick) were stained with 1% methylene blue in 1% borax and analyzed and photographed under a Nikon Eclipse 80i light microscope.

### Methodological caveats.

Two methodological issues limit our ability to make precise absolute estimates of symbiont cell numbers. First, *Hodgkinia* and *Sulcia* have irregularly shaped tube-like cells when they are present in bacteriome tissue (17, 33), although we note that their shape seems to become much more spherical during migration to eggs (Fig. 2 and 4). This variation in cell shape could affect the accuracy of our estimates of *Sulcia* and *Hodgkinia* cell numbers (specifically, we might sometimes count the same cell twice), and therefore the *Sulcia/Hodgkinia* ratio, but we would not expect it to affect this ratio differently in different cicada species. Additionally, it is difficult to determine the precise age of the eggs we sampled, which could potentially affect the numbers of symbiont cells present in the symbiont ball. To keep our results as consistent as possible between cicada species, we counted symbionts only in eggs where the symbiont ball was still apparently intact. This roughly corresponds to eggs that have been laid but in which the embryo had not yet begun to visibly develop.

### Data availability.

The amplicon sequencing data have been deposited in GenBank, under BioProject accessions PRJNA475285, PRJNA475287, and PRJNA476567.[Supplementary-material tabS2]

10.1128/mBio.02104-18.4 TABLE S2 Accession numbers: amplicon sequencing data. Download Table S2, XLSX file, 0.1 MB. Copyright © 2018 Campbell et al. 2018 Campbell et al. This content is distributed under the terms of the Creative Commons Attribution 4.0 International license.
