# Supplementary figures and images for: Changes in Endosymbiont Complexity Drive Host-Level Compensatory Adaptations in Cicadas
Source: mBio. 2018 Nov 13;9(6):e02104-18. doi: 10.1128/mBio.02104-18 (PMC6234865; doi:10.1128/mBio.02104-18)

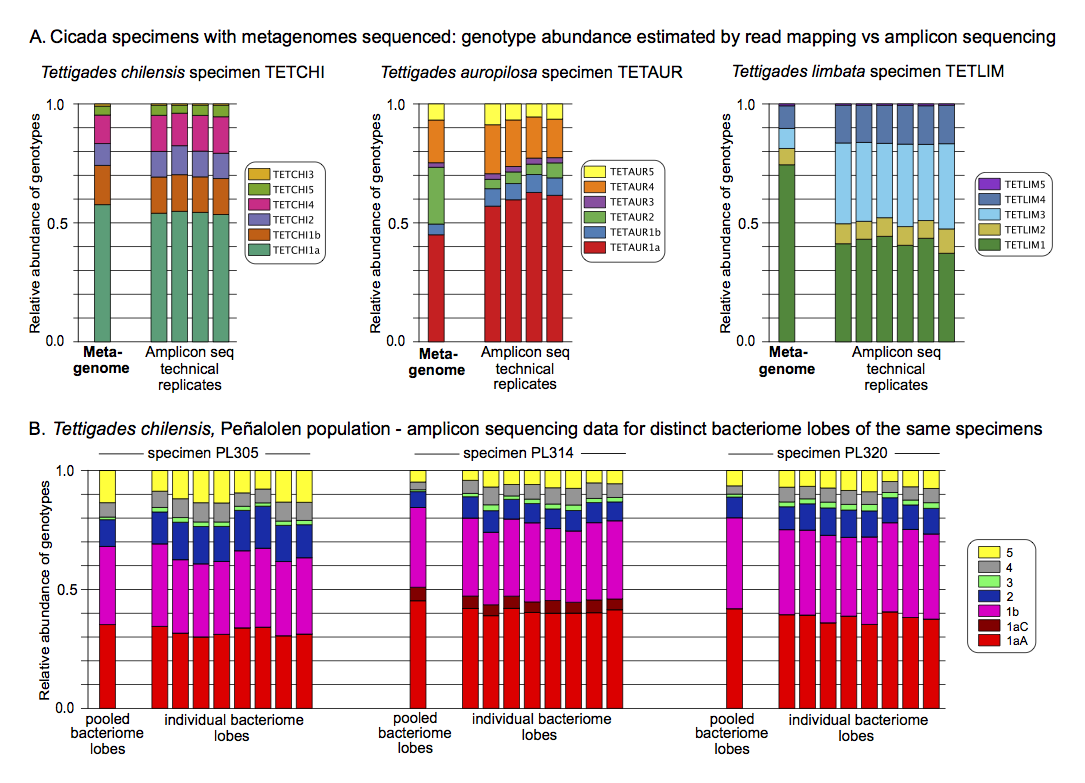

Supplement: FIG S1 [file mbo006184165sf1.tif]

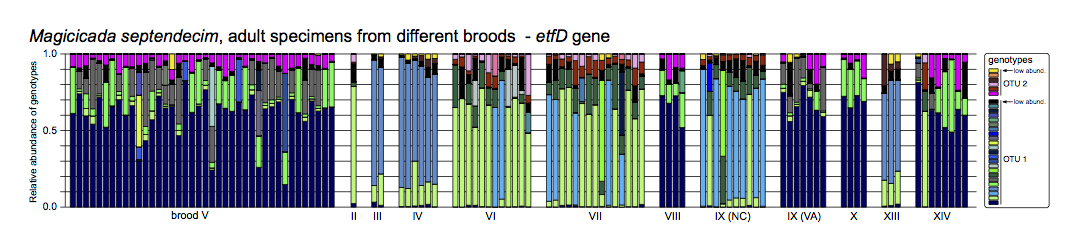

Supplement: FIG S2 [file mbo006184165sf2.tif]
